# Mouse Lymphoblastic Leukemias Induced by Aberrant *Prdm14* Expression Demonstrate Widespread Copy Number Alterations Also Found in Human ALL

**DOI:** 10.3390/cancers4041050

**Published:** 2012-10-18

**Authors:** Stephen J. Simko, Horatiu Voicu, Brandi L. Carofino, Monica J. Justice

**Affiliations:** 1 Baylor College of Medicine, Department of Pediatrics, Texas Children’s Cancer and Hematology Centers, One Baylor Plaza, Houston, TX 77030, USA; 2 Baylor College of Medicine, Dan L. Duncan Cancer Center, One Baylor Plaza, Houston, TX 77030, USA; E-Mails: voicu@bcm.edu (H.V.); mjustice@bcm.edu (M.J.J.); 3 Baylor College of Medicine, Department of Molecular and Human Genetics, One Baylor Plaza, Houston, TX 77030, USA; E-Mail: carofino@bcm.edu; 4 Baylor College of Medicine, Interdepartmental Program in Translational Biology and Molecular Medicine, One Baylor Plaza, Houston, TX 77030, USA

**Keywords:** PRDM14, lymphoblastic leukemia, pluripotency, CGH, leukemia, DNA damage, DNA repair, CDKN2A, TBX2

## Abstract

Aberrant expression and activation of oncogenes in somatic cells has been associated with cancer initiation. Required for reacquisition of pluripotency in the developing germ cell, PRDM14 initiates lymphoblastic leukemia when misexpressed in murine bone marrow. Activation of pluripotency in somatic cells can lead to aneuploidy and copy number alterations during iPS cell generation, and we hypothesized that PRDM14-induced lymphoblastic leukemias would demonstrate significant chromosomal damage. High-resolution oligo array comparative genomic hybridization demonstrated infrequent aneuploidy but frequent amplification and deletion, with amplifications occurring in a 5:1 ratio with deletions. Many deletions (*i.e*., *Cdkn2a*, *Ebf1*, *Pax5*, *Ikzf1*) involved B-cell development genes and tumor suppressor genes, recapitulating deletions occurring in human leukemia. Pathways opposing senescence were frequently deactivated via *Cdkn2a* deletion or *Tbx2* amplification, with corollary gene expression. Additionally, gene expression studies of abnormal pre-leukemic B-precursors showed downregulation of genes involved in chromosomal stability (*i.e*., *Xrcc6*) and failure to upregulate DNA repair pathways. We propose a model of leukemogenesis, triggered by pluripotency genes like *Prdm14*, which involves ongoing DNA damage and failure to activate non-homologous end-joining secondary to aberrant gene expression.

## 1. Introduction

Comprehensive analysis of the mutational landscape of cancer has provided significant insight into mechanisms of cancer initiation and maintenance. In acute lymphoblastic leukemia (ALL), the most common malignancy of childhood, patients have long been known to have characteristic translocations including *ETV6-RUNX1*, *TCF3-PBX1*, *BCR-ABL*, and rearrangements of MLL with numerous partners. With the exception of *BCR-ABL*, however, these translocations alone are not sufficient to initiate leukemia. Single nucleotide polymorphism (SNP) array analysis of mutations in childhood ALL has identified recurring deletions of B-cell development genes, including *IKZF1*, *PAX5*, and *EBF1* [1]. These data have subsequently been used to demonstrate evidence of an ancestral clone that differs from the primary diagnostic clone [2] and identify new markers of poor prognosis [3]. These abnormalities buttress our understanding of ALL as a genetically heterogeneous malignancy.

The specific developmental processes that lead to the formation of gene mutations, amplifications, and deletions in human lymphoblastic leukemias remain unclear. Given the normal role of V(D)J recombination in lymphoid development, inappropriate targeting of RAG1 and RAG2 has long been suspected in lymphoid leukemia initiation. RAG1/RAG2 recognize characteristic recombination signal sequences (RSS) in conjunction with adjacent H3K4 trimethylation [4]. Though RSSs are concentrated in immunoglobulin and T-cell receptor loci, they are found throughout the genome and can be inappropriately recognized, leading to inappropriate DNA deletion [5]. However, *Rag1*^−/−^ mice with concomitant deletion of the tumor suppressor locus *Cdkn2a* also develop lymphoid leukemias, implying that mechanisms of lymphoid leukemia development are not entirely dependent on mistargeted V(D)J recombination [6]. B-cell differentiation block contributes, but is not sufficient, to induce leukemia. *Ebf1* and *Pax5* deletions, though not sufficient to induce leukemia of their own accord, can each induce leukemia in mice with constitutive STAT5b activation [7]. Chromosomal instability also contributes to lymphomagenesis. Induction of chromosomal instability by disruption of DNA repair mechanisms, including ATM-dependent cell cycle checkpoints and telomere function, leads to murine T-lymphoblastic lymphomas with copy number alterations (CNAs) analogous to those seen in human disease [8].

The ability of gene misexpression to trigger chromosomal instability, induce CNAs, and initiate cancers is well characterized [9]. We have recently identified PR containing domain 14 (*Prdm14*) as a novel oncogene whose misexpression in hematopoietic precursors leads to B-cell differentiation block and, ultimately, lymphoblastic leukemia and lymphoma [10,11,12]. Little is known about the role of PRDM14 in cancer initiation and maintenance in humans, but its aberrant expression and role in chemoresistance in cancers has been noted. In addition to being overexpressed in T-ALL and hyperdiploid precursor B-ALL [12], *PRDM14* is amplified and misexpressed in breast cancers. Its silencing by RNAi results in increased chemosensitivity and decreased cell proliferation [13].

PRDM14’s role in cancer initiation may well be related to its role in inducing and maintaining a pluripotent state. PRDM14 is normally expressed only in cells of embryonic and germ cell lineage, and is not normally detected in other differentiated tissues [11,14]. Its expression is necessary for fertility; PRDM14 facilitates reacquisition of pluripotency and epigenetic reprogramming in germ cells after they migrate through the embryo to arrive at the primordial gonad [14]. PRDM14 does not accelerate pluripotency acquisition alone, but synergizes with other pluripotency factors. With KLF2, PRDM14 facilitates conversion of murine epiblast stem cells to embryonic stem cells, with concomitant X chromosome reactivation and DNA demethylation [15]. PRDM14 also plays a role in maintaining pluripotency, as it prevents embryonic stem cells (ESCs) from differentiating into extraembryonic endoderm [16]. In mouse and human ESCs, it binds genomic DNA at a twelve nucleotide consensus sequence and colocalizes with transcription factors necessary for pluripotency such as OCT4 (POU5F1), NANOG, and SOX2 [16,17], which in turn regulate PRDM14 expression [18,19]. Thus, while PRDM14 is not independently sufficient for pluripotency reprogramming, it does appear to accelerate and, in the developing germ cell, is necessary for this process to occur.

Activation of pluripotency in somatic cells has been associated with not only aneuploidy [20] but also CNAs. Addition of a *Myc*-containing vector to murine mammary cells to promote dedifferentiated mammosphere formation, or induction of pluripotency with *Oct4*, *Sox2*, and *Klf4* in murine embryonic fibroblasts, results in diploid induced pluripotency stem (iPS) cell formation that contain amplifications and deletions, particularly in common fragile sites of the genome [21]. Normal pluripotent cells, seemingly, would also be susceptible to DNA damage from activation of genes like *Myc*, except that they activate high-fidelity DNA repair mechanisms. For instance, iPS cells (like ES cells) upregulate expression of genes involved in DNA repair compared to more differentiated somatic cohorts [22]. In stem cells, PRDM14 maintains pluripotency [15] likely in the presence of DNA repair partners in order to maintain the integrity of the stem cell genome. In contrast, when *Prdm14* is misexpressed in somatic cells, DNA repair cofactors may be deregulated. Therefore, we hypothesized that expression of *Prdm14* in somatic cells causes cancer by activating self-renewal [12] without appropriate prevention of or response to DNA damage. The lack of appropriate repair may be evident as chromosomal aberrations or copy number alterations.

Here we use array comparative genomic hybridization (aCGH) to evaluate whether *Prdm14* expression in murine hematopoietic cells leads to tumors with substantial genomic derangements, compare CNAs to those seen in human disease, and explore possible mechanisms of DNA damage in these tumors.

## 2. Results and Discussion

We previously described PRDM14-induced development of lymphoblastic leukemia/lymphoma in mice transplanted with stem-cell enriched bone marrow transduced with a MIGR1-*Prdm14* vector (also referred to as MPr14 in tumor prefixes) [12]. In order to determine the downstream effect of constitutive *Prdm14* expression on the genome, we performed high-resolution (1 × 1 MB) array CGH to analyze 12 tumors of varying lymphoid cell lineage type for copy number gain and loss (Table 1). Tumors that arise from this model vary widely in lymphoid lineage, being primarily precursor-T or precursor-B, but some having a “mixed” phenotype based on presence of both IgH (or B-cell receptor, BCR) and T-cell receptor (TCR) rearrangement, or “common lymphoid progenitor” (CLP) based on lack of rearrangements. Further, some tumors showed evidence of an erthyroblastosis or megakaryocytosis. Tumors were classified according to presence of BCR/TCR rearrangement and were selected to reflect the variety of lineages [12].

**Table 1:** Characteristics of the tumors analyzed.

Sample #	Tumorlocation	IgHrearrangement	TCRrearrangement	Histological abnormalities	BCR/TCR-based tumor type
91 (control)	Spleen	N/A	N/A	None	N/A
92 (control)	Spleen	N/A	N/A	None	N/A
93 (control)	Spleen	N/A	N/A	None	N/A
MPr14-111	Lymph node	No	No	LL	Common Lymphoid Progenitor (CLP)-like
MPr14-143	Spleen	Yes	No	LL with erythroblastosis	Precursor-B
MPr14-147	Spleen	Yes	Yes	LL	Mixed
MPr14-148	Lymph node	No	Yes	LL with erythroblastosis	Precursor-T
MPr14-185	Lymph node	Yes	No	LL	Precursor-B
MPr14-189	Lymph node	Yes	No	LL	Precursor-B
MPr14-196	Spleen	Unknown	Unknown	LL with erythroblastosis	Unclassified
MPr14-197	Spleen	Yes	Yes	LL	Mixed
MPr14-217	Lymph node	Yes	No	LL	Precursor-B
MPr14-218	Lymph node	Yes	No	LL-pleiomorphic	Precursor-B
MPr14-228	Spleen	Yes	Yes	LL with increased megakaryocytes	Mixed
MPr14-258	Spleen	No	Yes	LL	Precursor-T

LL = lymphoblastic lymphoma. N/A = not applicable.

### 2.1. PRDM14-Induced Tumors Have Numerous, Recurrent Copy Number Alterations with Occasional Aneuploidy

**Figure 1:**
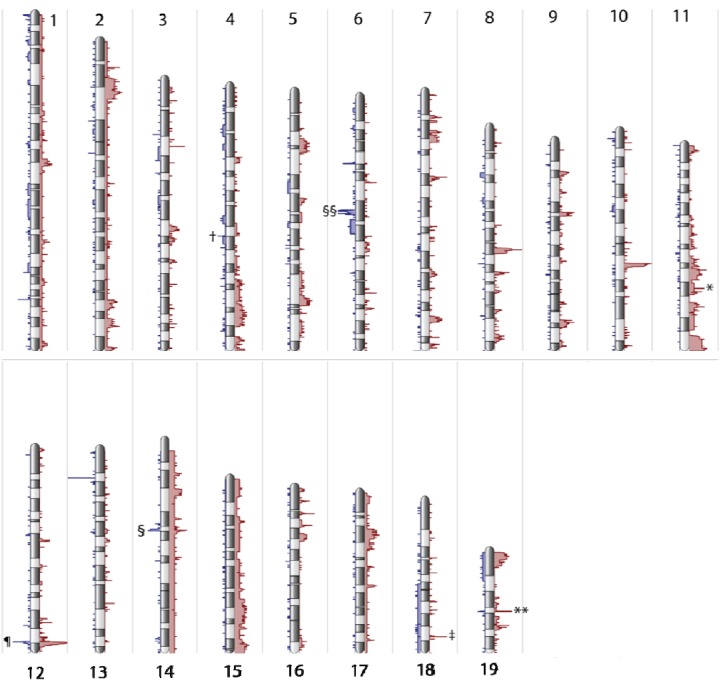
Global genome view histogram, depicting regions of amplification and deletion. X-axis depicts frequency of amplifications greater than 2.3 copies (red) or deletions less than 1.7 copies (blue). Chromosomes do not show frequent aneuploidy, though trisomy 14 and 15 are present in two tumors. Amplification of genes occurs more frequently than deletions in approximately a 5:1 ratio. Gene regions with common amplification and/or deletion include IgH locus (¶), T-cell receptor alpha (§) and beta (§§), *Cdkn2a* (†), *Sall3* (‡), *Tbx2* (*), and *Kif11* (**). Chromosomes X and Y reflect the sex of the tumor but demonstrate few CNAs and are depicted in Supplementary Figure 1.

Aneuploidy occurred at low frequency. Whole chromosome gains occurred in 4/12 tumors; one tumor had a partial deletion of chromosomes 18 and 19, but no other very large-scale (*i.e*., >10 Mb) CNAs were noted. Comparatively, CNAs occurred with high frequency (Figure 1), with amplifications much more common than deletions (Supplementary Table 1 and Supplementary Table 2). Excluding copy numbers affected by aneuploidy, 1,830 genes were amplified, and 378 genes were deleted across the 12 tumors. Tumors carried a mean of 152 amplifications and 31.5 deletions. A large fraction of CNAs were recurrent. We identified 168 unique recurring amplifications, spanning 439 genes, and 25 unique recurring deletions, spanning 35 genes. Median amplicon size was 91,543 bp, with a minimum amplicon size of 14,799 bp. Median size of deleted region was 99,798 bp with minimum size of 20,510 bp.

### 2.2. Deleted Genes Confirm Role of B-Cell Developmental Disruption in PRDM14-Induced Lymphomagenesis

**Figure 2:**
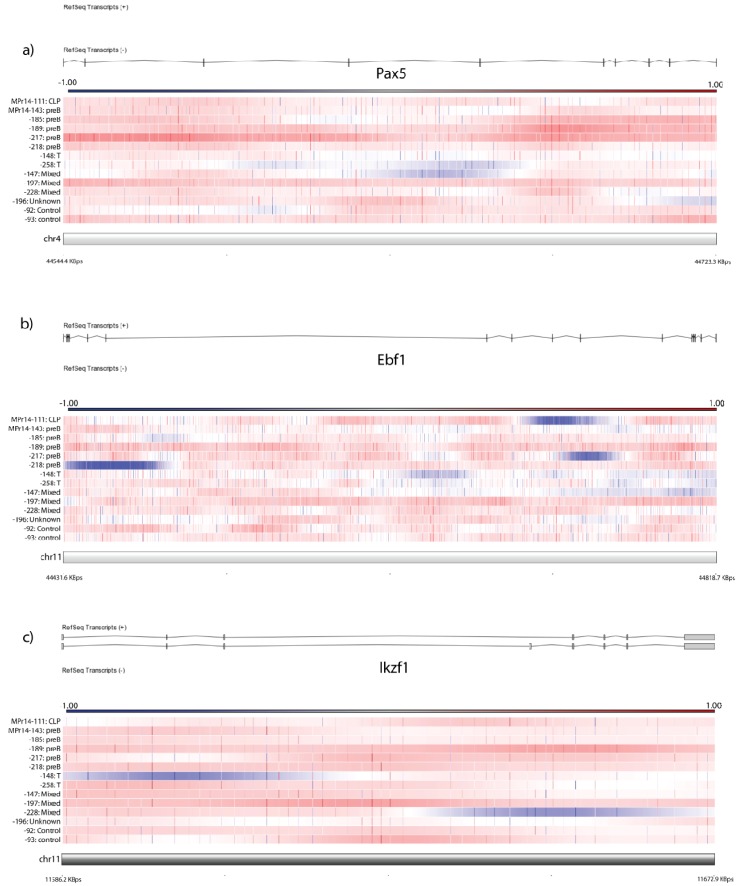
Copy number alterations in B-cell development genes. Horizontal bars reflect individual tumors; individual oligo amplification (red) and deletion (blue) are depicted by vertical marks, and areas of amplification/deletion are deleted by individual color change on the respective bars. Color code for CGH graphs is depicted above each graph and represents the log ratio of the array. Array depicted against exonic/intronic region of gene. Samples are arranged by BCR/TCR-based tumor type as listed in Table 1. Depiction of CNAs often cover small regions and are sometimes only intronic, as seen in (**a**) *Pax5*, (**b**) *Ebf1*, and (**c**)*Ikzf1*.

We previously reported B-cell differentiation block at the pro-B stage in mice transplanted with Prdm14-transduced bone marrow [12]. We evaluated tumors for deletions of genes involved in B-cell development and differentiation. Five of twelve tumors had deletions in critical B-cell developmental genes with tumor suppressor function, including *Ebf1*, *Pax5*, and *Ikzf1* (Figure 2a–c). Gene deletion of B-cell developmental genes occurred only in precursor-B lineage tumors, except in one tumor (MPr14-111) which did not have either B- or T-cell rearrangement, yet had the immunophenotype of a CLP-like tumor [12]. Numerous tumors have deletion within the immunoglobulin heavy chain and T-cell receptor region, consistent with rearrangement.

### 2.3. Deletions and Amplifications Occur in Characterized Tumor Suppressors and Oncogenes

**Figure 3:**
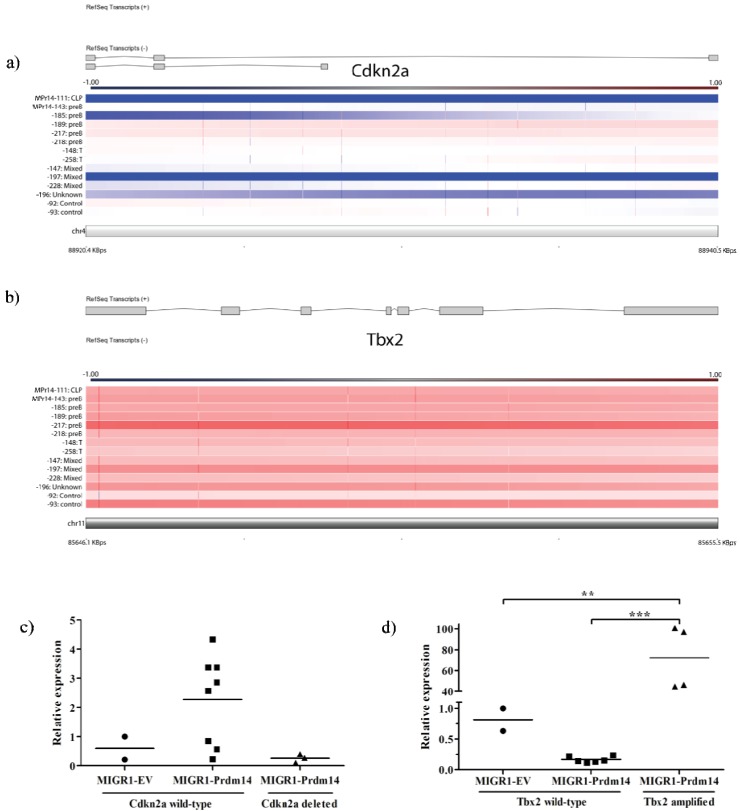
Depiction of regions of deletion and amplification, depicted with gene exons and gene expression. Color code for CGH graphs is depicted above each graph and represents the log ratio of the array (deletion is blue, amplification is red). Samples in (**a**) and (**b**) are arranged by BCR/TCR-based tumor type as listed in Table 1. (**a**) Four tumors have complete deletion of *Cdkn2a*, with entire sequence deleted.; (**b**) *Tbx2* is amplified in four tumors; (**c**) Expression of *Cdkn2a* is low in all tumors with gene deletion, but low expression is not exclusive to these tumors (*p* = not significant); (**d**) *Tbx2* expression is markedly elevated in all tumors with gene amplification. Amplification of *Tbx2* is reported in control #93 but no concomitant rise in expression is seen. *** indicates *p* < 0.001, ** indicates *p* = 0.001–0.01.

All tumors had at least amplification of one known oncogene or deletion of one tumor suppressor gene (Table 2). Given the expansion of abnormal lymphoid progenitors prior to leukemia onset, we expected to find derangement of apoptosis control genes. Four of twelve tumors had deletions of *Cdkn2a*, which encodes the INK4-ARF master apoptosis control proteins; gene expression was relatively low in all tumors with gene deletion, but expression across tumors does not appear to be solely dependent on gene deletion (Figure 3a,c). Four of twelve tumors had amplification of *Tbx2*, a gene involved in suppression of senescence. Though gene amplification appears to be heterozygous (mean copy number change of 3.02), gene expression is massively expanded in these tumors, with 45–101 fold change in gene expression in tumors with amplification (Figure 3b,d) compared to controls and tumors without amplification. Of note, one control appears to have gene amplification but, unlike the tumors, does not have increased gene expression.

**Table 2:** Tumor cytogenetics and major copy number amplifications.

Tumor #	Cytogenetics	BCR/TCR-based tumor type	Oncogene amplifications	Tumor suppressor deletions	Fragile site gene CNA	MLL rearrangement partners amplified
MPr14-111	40,XX	CLP-like		*Ebf1*, *Cdkn2a*		*Lpp*
MPr14-143	40,XX	Precursor-B	*Eg5*			
MPr14-185	43,XY,+14,+15,+17	Precursor-B	*Eg5*, *Tbx2*	*Cdkn2a*		*Mllt10*
MPr14-189	40,XY,del(18qD1→qter), del(19qter→qB)	Precursor-B	*Tbx2*			*Mllt10*, *Ell*, *Mllt3*
MPr14-217	40,XY	Precursor-B	*Notch1*, *Tcf3*, *Tbx2*	*Ebf1*	*Grid2*	
MPr14-218	41,XY,+1	Precursor-B		*Ebf1*	*Wwox*	*Ell*
MPr14-148	40,XX	Precursor-T	*Eg5*			
MPr14-258	40,XX	Precursor-T	*Eg5*			*Ell*, *Eps15*, *Lpp*
MPr14-147	42,XX,+14,+15	Mixed	*Eg5*	*Pax5*	*Grid2*	*Mllt10*
MPr14-197	40,XY	Mixed	*Mycl1*, *Eg5*, *Tbx2*	*Cdkn2a*, *Fhit*	*Fhit*	*Mllt10*, *Lpp*
MPr14-228	41,XY,+2	Mixed	*Lmo1*	*Ikzf1*	*Wwox*	*Mllt10*, *Ell*, *Lpp*
MPr14-196	40,XY	Unclassified	*Lmo1*, *Eg5*	*Cdkn2a*		

Seven of twelve tumors had amplification of *Kif11* (*Eg5*), a necessary component of the mitotic spindle required for proper chromosomal segregation, and whose amplification leads to the development of lymphomas and solid tumors in mice, usually associated with tetraploidy [23]. All tumors with erythroblastosis, an unusual feature in lymphoblastic leukemia and lymphoma, carried *Eg5* amplification, but not all tumors with *Eg5* amplification demonstrated this phenotype.

Five of twelve tumors have CNAs at known common fragile sites, genomic regions susceptible to DNA damage under replication stress [24]. Deletion of *Fhit* was detected in one tumor, and amplification of *Wwox* and *Grid2* was seen in two tumors each. Additional recurrent gene deletions occurred in genes spanning large (>200,000 kB) regions of DNA, including *Macrod2*, *Pcdh15*, and *Shroom3*.

### 2.4. T-lineage Lymphoblastic Leukemia/Lymphoma Demonstrate Fewer Candidate Driver Mutations than Precursor B or Mixed Lineage Leukemias

Leukemias were classified into subtypes based on presence of IgH or T-cell receptor rearrangements, and CNAs were analyzed by class. Principal component analysis demonstrated that leukemias did not group according to their condition, demonstrating no consistent global genetic changes that were class-defining. Although all classes of malignancies exhibited similar numbers of CNAs, we noted substantially fewer known leukemogenic CNAs in T-lymphoblastic leukemias than in other types (Table 2). As expected, B-cell development gene deletions (*i.e*., *Pax5*, *Ikzf1*, *Ebf1*) did not occur in T-lymphoblastic leukemias but did occur in other types (precursor B, CLP-like, mixed lineage). Deletions in common fragile sites, CNAs in *Cdkn2a* or *Tbx2*, and chromosomal rearrangements were also not seen in the T-lymphoblastic leukemias, although *Eg5* amplification was present. In comparing PRDM14-induced T-ALL mutations to previously published candidate driver mutations in murine T-lymphoblastic lymphoma identified using a Sleeping Beauty transposon mutagenesis screen [25], little overlap was noted, except for amplification of *Chst11* (tumor MPr14-148) and *Picalm* (tumor MPr14-258). Mixed/unclassified lineage leukemias demonstrated similar chromosomal changes as precursor-B leukemias, except for two tumors with amplification of *Lmo1*, a gene implicated in T-ALL leukemogenesis [26,27].

### 2.5. Gene Amplifications and Deletions Occur at PRDM14 Binding Loci

PRDM14-binding loci in murine ES cells have been previously characterized using ChIP-Seq pulldown of FLAG-PRDM14 [16]. We cross-referenced these known binding sites with genes amplified and deleted in PRDM14-induced tumors. Five of 35 genes (*Ebf1*, *Sox2ot*, *Shroom3*, *Slc10a7*, and *Ncam1* [which encodes CD56]) with recurrent deletions had PRDM14 binding sites within the gene region, and four of five had multiple binding sites. PRDM14 binds also within intronic sequence of *Pax5* [16], which is deleted in tumor 147 (Figure 2b). Though PRDM14 does not bind at a preponderance of amplified loci, it does bind at numerous amplified loci previously characterized in oncogenesis, including *Kif11*, *Lmo1*, *Notch1*, and *Lpp*.

### 2.6. Extensive Amplification of Known Cancer Rearrangement Partners

Rearrangements of the MLL gene with numerous partners in human leukemias have been characterized [28]. Though we were limited from performing karyotypic analysis of our tumors due to tissue availability, we did note substantial amplification of MLL rearrangement partners. Eight of twelve tumors demonstrated amplification of these genes, including *Lpp*, *Mllt3*, *Mllt10*, *Ell*, and *Eps15* (Table 2).

### 2.7. Genes Involved in Maintaining Chromosomal Stability Have Decreased Expression Prior to Leukemia Formation, and DNA Damage Response Fails to Activate

We previously described abnormal gene expression seen in an expanded population of common lymphoid progenitors (Il7ra^+^lin^−^Kit^+^Sca1^+^) that predated the onset of leukemia in our mice [12]. We performed gene ontology analysis from this experiment to evaluate for enrichment of genes involved in DNA repair that may be up- or downregulated. All statistically significant (p-value corrected for multiple testing <0.05) gene changes were evaluated, regardless of fold change (FC). Enrichment for genes involved in DNA repair was noted (*p* = 2.8E-2, Benjamini = 3.9E-1). Fourteen genes involved in DNA repair—*Xrcc6*, *Slk*, *Uhrf1*, *Usp1*, *Apbb1*, *Ddb1*, *Rad23b*, *Blm*, *Fancd2*, *Lig3*, *Brip1*, *Cinp*, *Ercc3*, and *Rtel1*—had statistically significant decreased expression (Supplementary Table 3). In particular, *Xrcc6* (FC = −3.31), *Fancd2* (FC = −1.27), *Blm* (FC = −1.26), and *Brip1* (FC = −1.23) are involved in maintaining chromosomal stability [29,30,31]. Of the ten upregulated genes—*Ube2b*, *Ube2a*, *Setx*, *Chaf1b*, *Ercc5*, *Mbd4*, *Poll*, *Mgmt*, *Msh5*, and *Cep164*—involved in DNA repair, only *Cep164* (FC = 2.0) is directly involved in maintenance of chromosome stability [32]. None of the other increased genes showed fold change above 1.4, and their role in DNA repair is restricted to nucleotide excision repair or protein ubiquitinization after radiation exposure [33]. None of the upregulated genes participate in homologous recombination repair (HRR) or non-homologous end-joining (NHEJ). Correlation with previously published PRDM14 binding within mouse ES cells demonstrated no PRDM14 binding within 100 kB of any of these loci [16].

Meiosis-specific genes (gene ontology analysis, *p* = 1.9E-1, Benjamini = 8.1E-1), including *Rsph1*, *Spo11*, *Zfp318*, *Msh5*, *Clgn*, and *Ube2b* are also upregulated in the abnormal pre-leukemic cells (Supplementary Table 3). Two genes with a known role in normal homologous recombination in meiosis are upregulated. *Msh5*, noted above, also is required for crossing-over in meiosis-specific DNA recombination [34]. *Spo11* induces double stranded breaks during meiotic recombination [35] and is upregulated 2.3 fold.

### 2.8. Discussion

PRDM14-induced lymphoblastic leukemia/lymphomas show heterogeneity of genomic aberrations, similar to that seen in human disease. Human ALL is characterized not just by genetic translocations but by sporadic deletions in genes necessary for B-cell development and differentiation (*Pax5*, *Ebf1*, *Ikzf1*) or apoptosis (*Cdkn2a*), as are seen frequently in our tumors. Unlike human ALL, however, PRDM14-induced leukemias bear many more gene deletions and particularly amplifications per individual. In their analysis of 228 ALL patients samples, Mullighan *et al*. reported an average of 3.83 deletions per individual ALL specimen, and only rare focal amplifications, arguing against genomic instability as a mechanism of cancer initiation in most childhood lymphoblastic leukemias [1]. Murine PRDM14-induced leukemia/lymphomas have a mean of 152 amplifications and 31.5 deletions. The high number of detected CNAs did not appear to be an artifact of overly permissive CNA analysis; frequent amplicons (mean 18.4 per tumor) were detected even using excessively strict (copy number >3 or <1) criteria. This level of CNAs is unusual for oncogene-induced murine tumors [8].

DNA damage appears to result from replication stress and chromosomal instability. The high frequency of common fragile site CNAs (5/12 tumors) plus additional CNAs within large genes suggests significant endogenous replication stress. DNA damage at common fragile sites has been shown to precede CNAs at other locations when comparing pre-neoplastic tissue to malignant cells [36]. The high number of CNAs in our tumors, along with the decreased expression of genes required for DNA repair, suggests that chromosomal instability also plays a role in our model of leukemogenesis. Decreased expression of *Xrcc6*, which is greater than 3-fold reduced in pre-leukemic cells, appears to be a primary mechanism of DNA repair failure. *Xrcc6* encodes the Ku70 subunit of the Ku heterodimer, an essential component of the DNA-dependent protein kinase (DNA-PK) complex required for initiation of NHEJ in double stranded break repair [29,37].

Failure to complete NHEJ alone does not explain the degree of DNA damage, particularly regarding amplifications. Pluripotency reprogramming normally induces high fidelity DNA repair mechanisms, including increased expression of numerous HRR and DNA repair genes [22]. Unlike in ES or iPS cells, however, aberrant *Prdm14*-expressing lymphoid progenitors fail to upregulate HRR or NHEJ genes. Only one gene, *Cep164*, has increased expression in response to aberrant somatic *Prdm14* expression, as opposed to the more global changes seen in iPS or ES cells relative to more differentiated counterparts; additionally, this gene does not participate in direct DNA repair but contributes to cell cycle arrest in response to DNA damage [32]. Thus, the combined effect of decreased expression of DNA repair enzymes plus failure to upregulate DNA repair mechanisms in response to replication stress appears to be the critical mechanism of eventual widespread DNA damage.

**Figure 4:**
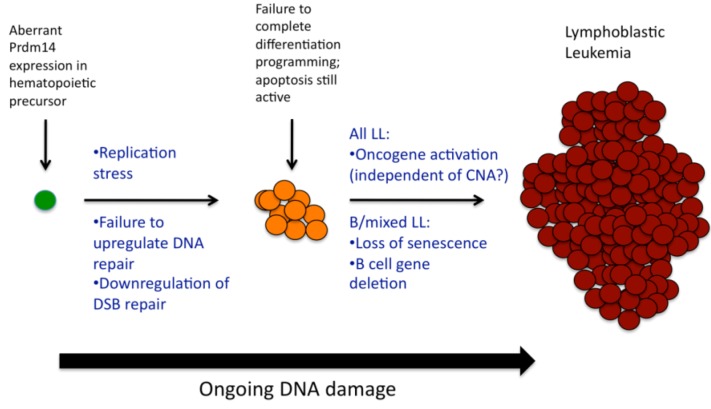
PRDM14 induces cancer via multifactorial mechanisms. Early changes involve failure of DNA repair mechanisms in the setting of replication stress, though apoptosis mechanisms are active [12]. Subsequent changes involve loss of senescence, deletion of B-cell genes, and/or activation of oncogenes.

Knowing that PRDM14-induced lymphoid progenitors have differentiation block at the pro-B stage [12], and knowing now that putative leukemia precursor cells have impaired ability to repair double-stranded breaks, a more complete model of *Prdm14*-related cancer initiation emerges (Figure 4), particularly as related to B-lineage or mixed B/T leukemias. PRDM14 expression attempts to reprogram differentiating hematopoietic precursors. The replication stress contributed by activation of pluripotency, the deactivation of genes involved in chromosomal stability (*Xrcc6/*Ku70), the failure to activate DNA repair mechanisms, and the activation of homologous recombination factors lead to excessive DNA damage. Cells fail to complete the normal B-cell differentiation program, perhaps due to impairment of NHEJ in V(D)J recombination or deletion of B-cell development genes. Furthermore, stem cell reprogramming does not occur properly, likely because other pluripotency factors are missing [15]. Later events include activation of oncogenes (*Lmo1*, *Notch1*) sometimes but not necessarily via amplification; we previously demonstrated that *Myc*, for instance, has increased expression in tumors but not in preleukemia, and is not amplified [12].

Tumors appear to have arisen only after the mutational burden and involvement of oncogenes and tumor suppressors circumvented highly active apoptosis pathways. Notably, our previous gene expression studies of pre-leukemic progenitor cells noted upregulation of multiple p53 pathway genes, including *p21* [12], suggesting that preleukemic cells appear to have intact apoptosis mechanisms. Ultimately, however, senescence mechanisms appear to be defeated in our tumors by genomic alteration. Six of twelve tumors demonstrate either amplification of *Tbx2* or deletion of the tumor suppressor *Cdkn2a*. It is deleted here (33%) at a similar rate as seen in human disease (25%) [1]. In human lymphoblastic leukemia, but not in solid tumors, its mechanism of deletion involves aberrant RAG targeting, as RSSs flank *CDKN2A* breakpoints [5]. TBX2 inhibits *CDKN2A* by repressing its promoter, preventing expression that would otherwise be induced by *Myc* or *Ras*-pathway genes [38]. *TBX2* also binds the promoter and represses expression of p21, a key mediator of senescence [39]. In our model, the abrogation of senescence and concomitant failure of DNA repair appears to be a critical mechanism of Prdm14-induced tumorigenesis.

Curiously, this model does not completely apply to TCR-rearranged leukemias without IgH rearrangements seen in this study. Though similar degree of genomic derangement is seen in these tumors, few CNAs in these tumors are in known oncogenes or tumor suppressors, or in candidate tumor initiating genes. *Kif11* (*Eg5*) is amplified in these tumors, but whether this represents a driver or passenger mutation is unknown, given the profound aneuploidy seen in EG5-induced tumors that is not seen these PRDM14-induced leukemias [23]. The mechanism of PRDM14-induced T-leukemogenesis remains a subject for further study.

Of the amplified loci with known PRDM14 binding sites, many of these involve genes implicated in oncogenesis. *LMO1* is amplified in a subset of human T-lymphoblastic leukemias [27], and its gene product works in concert with SCL to create conditions allowing NOTCH1 activation and T-lymphomagenesis [26]. A majority of tumors also have *Kif11 (Eg5)* amplification, which is known to induce tetraploid tumors in mice [23]. The frequency of these and other oncogene amplifications at PRDM14 binding loci, as well as the presence of recurrent deletions at or near PRDM14 binding sites, raises the question of whether PRDM14 binding contributes to replication stress by directly interfering with normal DNA replication mechanisms in somatic cells.

## 3. Experimental Section

### 3.1. Animal Care

All mouse experiments were carried out under the approval of the Institutional Animal Care and Use Committee at Baylor College of Medicine (BCM). Mice were housed in the barrier facility at BCM, under the care of the Center for Comparative Medicine, which is accredited by the Association for Assessment and Accreditation of Laboratory Animal Care International.

### 3.2. Transduction of Prdm14 into Stem-Cell Enriched Bone Marrow Cells

The protocol for transducing bone marrow with *Prdm14*-containing vector was described previously [12]. Briefly, donor CD45.2 C57BL/6J mice were injected with 5-fluorouracil five days prior to bone marrow harvest. Extracted marrow was transduced with a GFP-labeled murine stem cell virus-based vector (MIGR1) containing *Prdm14* (MIGR1-*Prdm14*) or empty vector (EV). Transduced marrow was transplanted into lethally irradiated CD45.1 C57BL/6J mice. Mice were aged until development of systemic illness; mice sacrificed for illness were noted to have lymphoblastic leukemia/lymphoma as described [12]. Four weeks after transplantation, a fraction of mice were harvested, and marrow cell suspensions underwent cell sorting to isolate cells with a common lymphoid progenitor signature (CLPs; Il7ra^+^lin^−^Kit^+^Sca1^+^; no additional sorting for GFP) as described [12].

### 3.3. Array CGH

Twelve leukemic lymph nodes and spleens from MIGR1-*Prdm14* transduced mice were selected for aCGH analysis (Table 1). Three spleens from EV-transduced mice were selected as controls. Genomic DNA was extracted from frozen, unsorted tumor samples using DNeasy Blood and Tissue Kit (Qiagen, Valencia, CA). Sample quality check and chip hybridization were performed by the BCM Genomic and RNA Profiling Core Facility. DNA underwent amino-allyl labeling with Cy3 (control) and Cy5 (samples) and was hybridized to Mouse aCGH SurePrint G3 (1 × 1 M) slides (Agilent, Santa Clara, CA, USA). Two control specimens also underwent Cy5 labeling for comparison to the third control specimen. Array was scanned using GeneTAC UC-4 Microarray Analyzer (Digilab, Holliston, MA, USA). Data were analyzed and visualized using Partek Genomic Suite (Partek, Chesterfield, MO, USA). Results were preprocessed using quantile normalization and were adjusted for identical distribution. Deletions and amplifications were evaluated by genomic segmentation, comparing copy number in samples versus control. The minimum genomic markers in any segment is 10, the *p*-value threshold for two neighboring regions having significantly different means is 0.001, and the minimum signal-to-noise ratio for each transition is 0.3. To correct for admixture of normal tissue within tumor tissue, and allowing for the possibility of several subclones of cells within the leukemia population, cutoffs of copy number greater than 2.5 or less than 1.5 were used to call amplification and deletion, respectively. As a control for this, a control of greater than three chromosomes or less than one failed to detect differences in sex chromosome genes.

### 3.4. Gene Expression Array

Gene expression array was performed on Il7ra^+^lin^−^Kit^+^Sca1^+^ cells as described previously [12]. Gene ontology analysis was performed using functional annotation clustering in the Database for Annotation, Visualization, and Integrative Discovery (DAVID) [33,40].

### 3.5. Gene Expression Studies

Total RNA was isolated from frozen control EV spleens and tumors using TRIzol Reagent (Life Technologies, Carlsbad, CA, USA). RNA was reverse transcribed using the SuperScript III First-Strand Synthesis System for RT-PCR kit (Life Technologies). Resulting cDNA was amplified using real-time PCR with Power SYBR Green PCR Master Mix (Life Technologies) and gene-specific primers (Supplementary Table 4). Amplification and data analysis were conducted on a Rotor-Gene Q machine and software (Qiagen, Valencia, CA, USA). Relative gene expression was calculated with the 2^−ΔΔCt^ method [41]. Expression of ribosomal protein L19 (Rpl19) was used as an endogenous control, and all values are relative to control EV spleen. Gene expression levels in tumors with amplification were compared to levels in tumors without amplification and in control EV spleen using a one-way ANOVA with Bonferroni correction for multiple comparisons. Statistical analysis and visualization of PCR results were conducted using Prism (GraphPad, La Jolla, CA, USA).

## 4. Conclusions

Aberrant *Prdm14* expression in somatic cells not only initiates cancer but also induces profound DNA rearrangements. These data emphasize the role of pluripotency activation and cellular reprogramming in cancer initiation, and particularly the role of transcription factors such as PRDM14 that accelerate but do not complete pluripotency reprogramming, and that do not co-activate appropriate DNA repair mechanisms.

## Supplementary Files

Supplementary File 1 ZIP-Document (ZIP, 1619 KB)
