# Supplementary material for: Mouse Lymphoblastic Leukemias Induced by Aberrant Prdm14 Expression Demonstrate Widespread Copy Number Alterations Also Found in Human ALL
Source: Cancers (Basel). 2012 Oct 18;4(4):1050–66. doi: 10.3390/cancers4041050 (PMC3593237; doi:10.3390/cancers4041050)

Supplementary Figure 1. Global genome view histogram of chromosome X (left) and Y (right), depicting regions of amplification and deletion. X-axis depicts frequency of amplifications greater than 2.3 copies (red) or deletions less than 1.7 copies (blue). Variance in Y chromosome (some samples with more, some with less Y chromosome) reflects mixed gender of donor cells in control tissue.

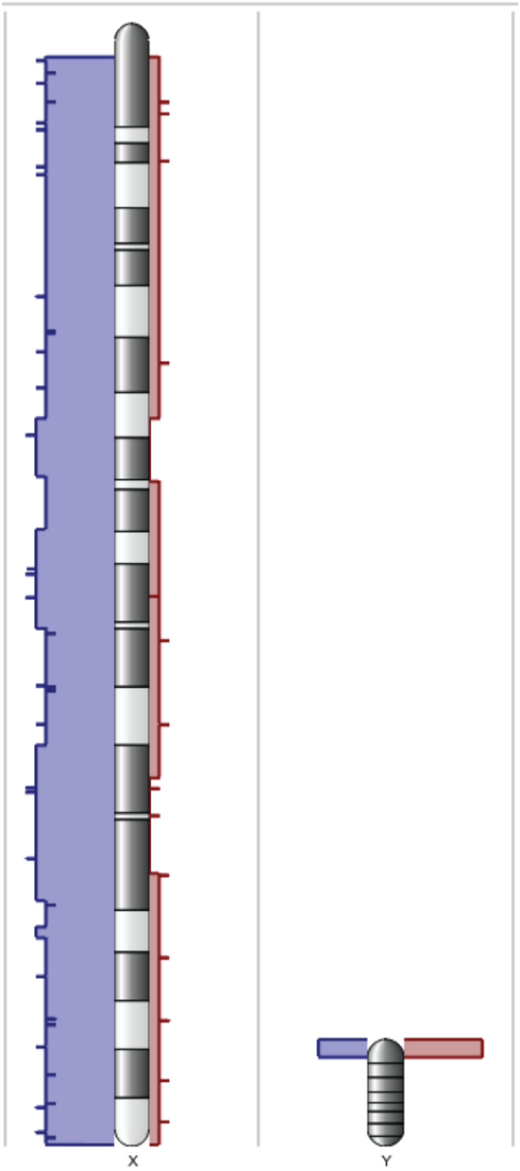

Supplement: Supplementary File 1 — ZIP-Document (ZIP, 1619 KB) [file cancers-04-01050-s001.zip › cancers-21444-supplement-final/Supplementary Figure 1.pdf]
