# Supplementary material for: Mouse Lymphoblastic Leukemias Induced by Aberrant Prdm14 Expression Demonstrate Widespread Copy Number Alterations Also Found in Human ALL
Source: Cancers (Basel). 2012 Oct 18;4(4):1050–66. doi: 10.3390/cancers4041050 (PMC3593237; doi:10.3390/cancers4041050)
Supplement: Supplementary File 1 — ZIP-Document (ZIP, 1619 KB) [file cancers-04-01050-s001.zip › cancers-21444-supplement-final/Supplementary Table 3.pdf]

Supplementary Table 3. Fold change in expression of genes involved in DNA repair or meiotic recombination. Illumina MG6 Chips were used to assess expression in MIGR1-Prdm14-induced pre-leukemic cells versus empty vector pre-leukemic cells [12]. Changes in those genes involved in DNA repair or meiosis are shown.

Gene symbol	Fold change	Adjusted P value corrected for multiple testing	Alternate gene symbol	Role
Rsph1	3.6191881	0.00270947	*Tsga2*	Meiosis
Spo11	2.260731973	0.00279317		Meiosis
Cep164	1.996555387	0.000529528	*D030051D21*	DNA Repair
Zfp318	1.7090723	0.017328638		Meiosis
Msh5	1.3772303	0.048407612		Meiosis
Mgmt	1.362312072	0.031249771		DNA Repair
Ercc5	1.362075071	0.027032501		DNA Repair
Poll	1.3441006	0.025402621		DNA Repair
Clgn	1.324112028	0.004936568		Meiosis
Mbd4	1.3031165	0.037776596		DNA Repair
Chaf1b	1.2656371	0.003541039		DNA Repair
Setx	1.219389	0.038398723	*AW060766*	DNA Repair
Ube2a	1.167635347	0.034720342		DNA Repair
Ube2b	1.149527262	0.04546337		Meiosis
Rtel1	−1.114172445	0.049838753	*AW540478*	DNA Repair
Ercc3	−1.199593813	0.043022755		DNA Repair
Brip1	−1.22568508	0.006826531		DNA Repair
Cinp	−1.243907244	0.024792337	*2810452K22Rik*	DNA Repair
Lig3	−1.249874087	0.046508525		DNA Repair
Blm	−1.25666748	0.005006649		DNA Repair
Fancd2	−1.256685205	0.027651204		DNA Repair
Rad23b	−1.271030696	0.034107433		DNA Repair
Ddb1	−1.27729361	0.039432097		DNA Repair
Apbb1	−1.295836127	0.007800335		DNA Repair
Usp1	−1.379884985	0.046051803		DNA Repair
Uhrf1	−1.412413922	0.009732253		DNA Repair
Slk	−1.949725994	0.000748194		DNA Repair
Xrcc6	−3.275334807	0.002152631	*G22p1*	DNA Repair
